# Evaluating the Effectiveness of the How to Talk to Your Doctor HANDbook Program

**DOI:** 10.3928/24748307-20190404-01

**Published:** 2019-05-31

**Authors:** Charleen McNeill, Lisa Washburn, Kristie B. Hadden, Zola Moon

## Abstract

**Background:**

Millions of Americans have low health literacy, potentially leading to a number of issues including medication errors, hospital admissions, unnecessary emergency department visits, skipped screenings and shots, and misinterpretation of treatment plans. People with low health literacy have less knowledge of illness management, less ability to share in decision-making, and poorer self-reported health status. Addressing health literacy is necessary to improve health care quality, reduce costs, and reduce disparities.

**Objective:**

The How to Talk to Your Doctor (HTTTYD) HANDbook Program addresses health literacy among rural participants who have low incomes, with a focus on improving health communication among populations that are medically vulnerable by using the HANDbook tool.

**Methods:**

Participants were recruited from 55 rural counties by county extension agents (CEA) to participate in the 1-hour HTTTYD session. Pre- and post-test surveys were completed. A subset of the sample completed a 3-month follow-up survey.

**Key Results:**

Of the 548 participants who fully completed the survey, a Wilcoxon Signed-Rank Test was performed on 484 of the participants who completed both the pre- and post-test. A statistically significant median increase in overall confidence among the participants from pre- (*M* = 15.99) to post-test (*M* = 17.76), (*z* = 13.454, *p* = .000), was noted. A subset of 166 participants also completed the 3-month follow-up survey. A significant increase in health literacy after participation in the HTTTYD HANDbook program from pre-test to 3-month follow-up was noted; effect sizes ranged from moderate to large.

**Conclusion:**

The HTTTYD HANDbook program meets recommendations for successful health literacy programs; significant positive outcomes demonstrate program effectiveness. HTTTYD HANDbook program delivery in rural communities by CEAs demonstrates access to understudied and often difficult-to-reach populations. **[*HLRP: Health Literacy Research and Practice*. 2019;3(2):e103–e109.]**

**Plain Language Summary:**

The How to Talk to Your Doctor HANDbook program delivered by county extension agents in rural communities showed capacity to access understudied and often difficult-to-reach populations. The significant, sustained improvement in health literacy noted among program participants demonstrated program effectiveness among those with low health literacy.

Health literacy is the degree to which one has the capacity to obtain, process, and understand basic health information and services needed to make appropriate health decisions (Parker, Ratzan, & Lurie, 2003). Over 80 million Americans have low health literacy, which can lead to medication errors, hospital admissions, unnecessary emergency department visits, skipped screenings and shots, and misinterpretation of treatment plans (Berkman, Sheridan, Donahue, Halpern, & Crotty, 2011). People with low health literacy have less knowledge of illness management, less ability to share in decision-making, and poorer self-reported health status (Kindig, Panzer, Nielsen-Bohlman, 2004). Addressing health literacy is necessary to improve health care quality, reduce costs, and reduce disparities (Kindig, Panzer, & Nielsen-Bohlman, 2004).

Interventions to improve health literacy in rural populations are particularly important due to the high prevalence of chronic disease and poor health indicators in these communities including high rates of obesity, hypertension, diabetes, and food insecurity (Zahnd, Scaife, & Francis, 2009). It is common for rural counties in the United States to be among those with the worst health status and the lowest levels of health literacy (Chesser, Burke, Reyes, & Rohrberg, 2016; Peng, Yuan, & Holtz, 2016; University of Arkansas Division of Agriculture, 2015). Rural community-based programs to address issues related to low health literacy are needed (National Academies of Sciences, Engineering, and Medicine, 2018), especially because rural residents often have more limited access to health care resources than their urban counterparts. The Cooperative Extension System, a nationwide network through the land grant university system, is uniquely positioned to increase access to such programs, particularly in rural areas. Extension professionals have long addressed local health issues through education and outreach, but it was not until 2014 that the National Framework for Health and Wellness was established and included health literacy as one of seven major focus areas. This document stated that “the same system of Extension can do for the nation's health what it did for American agriculture” (Braun et al., 2014, p. 2).

In response to health literacy concerns, each of the 50 states has created for its own citizens programs intended to improve health literacy (Centers for Disease Control and Prevention, 2018). Evidence is unavailable regarding program process or effectiveness despite the importance of community-based interventions to improve health literacy and communication with health care providers (Berkman, Sheridan, Donahue, Halpern, & Crotty, 2011; Berkman, Sheridan, Donahue, Halpern, Viera, et al., 2011; National Academies of Sciences, Engineering, and Medicine, 2018). Although a limited number of programs to improve health literacy among older adults exist, more evidence-based interventions to meet health literacy gaps in specific populations, including older adults and rural residents, are needed (Manafo & Wong, 2012; National Academies of Sciences, Engineering, and Medicine, 2018). Extant programs like Ask Me 3 (Institute for Healthcare Improvement, 2018) have established effectiveness in terms of facilitating discussion with health care providers but are not well known (Lapiz-Bluhm, Weems, Rendon, & Perez, 2015). Effective community-based health literacy interventions are critical to meeting health literacy goals.

Health literacy has different dimensions including reading, comprehending, and communicating medical and health information (Parker et al., 2003). Health literacy and communication are critical components to improve a person's overall health and the quality of care he or she receives (Office of Disease Prevention and Health Promotion, 2018). The project described here addresses health literacy among rural participants who are low-income by using the How to Talk to Your Doctor (HTTTYD) HANDbook Program. The HANDbook was the primary tool used in educational sessions teaching participants strategies for improving communication with health care providers. This article briefly describes the HTTTYD HANDbook program delivered through the Cooperative Extension Service in a rural state, then presents findings from participant data.

## HTTTYD Program

The HTTTYD HANDbook program was developed and field tested as a single-session program designed for delivery in community-based settings by health educators. HTTTYD sessions typically lasted for 1 hour and were delivered by county Extension educators and trained volunteers in various settings (churches, community centers, county Extension offices). Site selection is key as the National Academies of Sciences, Engineering, and Medicine (2018) highlights the importance of conducting community-based health literacy interventions in safe and comfortable locations known to the community.

The HTTTYD HANDbook includes five steps to prepare a person for health care provider appointments. Each step is represented by an individual finger on a hand: (1) remember the things you need to take to your visit; (2) two-minute history: tell your doctor your health problems in 2 minutes; (3) words: repeat instructions and information back in your own words; (4) FORms: don't forget to fill out all your FORms completely; and (5) take your meds: take medications as labeled and take all your medications to your doctor's visit. Teaching techniques used modeling, scenarios, role-play, and Teach Back, which provided participants opportunities to practice new communication skills. Examples of recommended strategies included getting assistance to make a list of questions to take to appointments, writing down prescriptions to take to appointment, writing down reasons for the visit and changes in health status, taking notes during the visit, and requesting written instructions for medicine and treatments. Sessions were designed to minimize confusion and eliminate barriers common for audiences with low literacy. For example, one piece of paperwork was passed out at a time (i.e., informed consent letter, pre-class questions), and question and answer response options were read aloud. HTTTYD HANDbook sessions were most often delivered in small group settings allowing for interactions between participants and trainers. Readability assessment of the HTTTYD Handbook revealed a mean level of 4th grade (Flesch, 1948; Fry, 1977; McLaughlin, 1969).

## Methods

This study was designed to answer two fundamental research questions: “What changes were noted in participants' confidence in engaging in communication with their doctors after participation in the HTTTYD HANDbook' program?” and “Were those changes sustained after 3 months?”

### Data Collection

After Institutional Review Board Approval at the University of Arkansas, participants were recruited from 55 rural counties by County Extension Agents (CEA) by advertising and hosting their own programs or through existing groups at established sites (i.e., senior centers). Participants completed a sign-in slip to document attendance and indicated if they gave permission for a member of the research team to contact them in 3 months to ask follow-up questions. Criteria for participation in the study included written consent from the participant, older than age 18 years, and able to read and write in English at a basic level.

The first question was a validated health literacy screening question, “How confident are you at filling out medical forms by yourself?” (Stagliano & Wallace, 2013). Four additional questions were developed by two health literacy experts and a health promotion expert. These questions were framed by the steps in the HTTTYD HANDbook, measuring change in confidence for each HANDbook step before and after the intervention. The questions were:

1. “How confident are you at being prepared to talk to your doctor?”
2. “How confident are you at telling your doctor everything he or she needs to know in a short visit?”
3. “How confident are you at repeating back what your doctor tells you in your own words?”
4. “How confident are you at taking your meds like the label says to?”

These questions were asked at three different time points: the beginning of the session to establish a baseline, the end of the session to measure immediate impact of the intervention, and at 3 months to measure sustainability of changes. All participants agreeing to participate in the research were administered pre- and post-test questions. Those participants who also agreed to follow-up surveys to were contacted 3 months later using their indicated preferred communication method (phone, mail, or email). Nonrespondents were contacted using their preferred method 3 times, each 1 week apart.

Readability of the questions was assessed using three formulas, each yielding a grade level result: Flesch-Kincaid, FORCAST, and Fry Readability. The mean readability result for the questions was a 4.8 grade level, which is interpreted to be “easy to read.” For studies using participants with low literacy, the reading level should be at or below the 5th or 6th grade level (Hersch, Salzman, & Snyderman, 2015).

### Sample

Fifty separate training sessions were held in 33 of the 55 targeted counties. Approximately 75% of the training was conducted by 21 CEAs. Five hundred and forty-nine participants completed the pre- and post-tests. One survey was disregarded due to missing a properly signed consent form. The final sample for the pre- and post-analysis was 548. Most of the participants were female (*n* = 460), White (*n* = 362), older than age 65 years (*n* = 362), and from a rural area (*n* = 417) (**Table 1**).

### Analysis

All data analyses used SPSS version 25. Descriptive statistics summarized demographic data and quantification of health literacy items. A sum scale was calculated using the four Likert-style questions that were listed earlier in the article. All questions were reverse-scored so the 5-point Likert scale would assign a value ranging from 5 for *extremely confident* and 1 for *not at all confident*. Potential scores for the sum scale range from 4 to 20.

## Results

No statistically significant differences were found between counties, training locations, or trainers when participants' age, gender, or race were analyzed. The data did not meet the assumptions of normality; therefore, a Wilcoxon Signed-Rank Test was performed on 484 participants who completed both the pre- and post- test. Of those participants, the HTTTYD HANDbook Program improved participant confidence among 296 (61.2%) participants, whereas 45 (9.3%) participant scores decreased from pre- to post-test, and 143 (29.5%) participant scores remained unchanged. The HTTTYD HANDbook program elicited a statistically significant mean increase in overall confidence among the participants from pre- (*M* = 15.99) to post- test (*M* = 17.76), (*z* = 13.454, *p* = .000). See **Table 2** for details on individual question analysis for the pre- and post-tests.

Of the 548 participants, 166 completed the 3-month follow-up survey (**Table 1**). Comparison of the repeated measures was performed using Friedman's test for each of the pre-, post-, and 3-month follow-up questions for this subset of participants. The pre- to post-testing score increased on each of the health literacy questions. A slight decrease in mean score was found from post-test to 3-month follow up for confidence in repeating back what one's doctor tells them in his or her own words as well as the overall mean scores. All other scores increased from post-test to 3-month follow up (**Table 2**).

A Wilcoxon Signed Ranks test with a Bonferroni correction was performed for each pre-, post-test, and 3-month follow-up question to determine at which points and in which direction significant changes occurred. As indicated in **Table 2**, a significant increase from pre- to post-test for each of the four questions and the overall score is evident. A Wilcoxon Signed Ranks test with a Bonferroni correction was performed for each pre- and 3-month follow-up question to determine whether the changes noted in the program were sustained at the 3-month follow-up period. No significant decreases in scores from post-test to the 3-month follow up for any of the questions were found except one: confidence in repeating back what one's doctor says in his or her own words. However, statistically significant increases in health literacy in each area and overall scores were sustained from pre-test to 3-month follow-up with effect sizes ranging from moderate to large (**Table 2**).

## Discussion

Of primary importance in this study is the sustained improvement in health literacy after participation in the program. Improvements were noted at the post-test and at the 3-month follow up. Previous studies show mixed results for other health literacy interventions (Berkman, Sheridan, Donahue, Halpern, & Crotty, 2011; Berkman, Sheridan, Donahue, Halpern, Viera, et al., 2011; Jacobs, Lou, Ownby, & Caballero, 2014). Much of the previous research evaluated interventions focused on specific topics like disease self-management, treatment regimen adherence, or changes to intervention design features (Mcluckie, Kutcher, Wei, & Weaver, 2014; Zullig, McCant, Melnyk, Danus, & Bosworth, 2014). Poor design of the studies, small sample sizes, and/or inconsistent results call this evidence into question (Berkman, Sheridan, Donahue, Halpern, & Crotty, 2011; Berkman, Sheridan, Donahue, Halpern, Viera, et al., 2011; National Academies of Sciences, Engineering, and Medicine, 2018). A novel aspect of the HTTTYD HANDbook program lies in its generalized approach, facilitating a better overall understanding of how to communicate with health care providers. Improving communication skills may result in participants more effectively relaying their health concerns and needs.

Despite sustained improvements in each skill from pre-test to 3-month follow-up, one question, confidence in repeating back what one's doctor tells them in his or her own words, did not demonstrate the same sustained improvement. Doctor-patient communication impacts compliance, patient satisfaction, and ultimately health (Aelbrecht et al., 2014; Matusitz & Spear, 2014), and many research endeavors have focused on the identification of problematic communication and methods to improve communication (Matusitz & Spear, 2014). Communication difficulties between doctors and patients can arise from lack of a shared understanding, lack of patient-centeredness, issues with confidentiality and trust, the number of interruptions, nonverbal communication, gaps in cultural understanding, and other issues (Matusitz & Spear, 2014). Research to better understand why patients may not be able to repeat what their doctor tells them is needed.

Use of participatory methods in HTTTYD HANDbook development enhanced the quality and the appropriateness of the program (Wilsher, Brainard, Loke, & Salter, 2017). Despite the potential value of such participatory methods, few health literacy studies selected these methods (Wilsher et al., 2017). The HTTTYD HANDbook program and materials development and implementation included an iterative process of prototype development, plain language editing, and field testing with a focus group of eight community members who provided guidance on several aspects of the program including the title, organization of information, understandability of content, actionability of content, and style preferences. A focus on skill development (i.e., preparing for appointments by making a list of questions, practicing a 2-minute history, writing down prescriptions and over-the-counter medications, and writing down reasons for the visit and changes in health status in preparation for an appointment) may help participants improve their confidence in communicating with health care providers. Improving patient communication skills could improve adherence to treatment regimens, although future research is needed.

Another important aspect of the program is that sessions were delivered by university-affiliated CEAs and trained volunteers. Both groups are trusted, credible community members (National Academies of Sciences, Engineering, and Medicine, 2018). Because education and outreach services are often limited or nonexistent in rural areas, using the existing land grant university system and network of the Cooperative Extension is beneficial. The Extension System is an important but underutilized, and often unrecognized, community resource for patients and the health care delivery system. Strengthened connections between health care providers and Extension as a source of health outreach and education would benefit patients and providers. As evidenced by the sustained changes in this study and the moderate to large effect sizes, Extension is an effective partner to address local health literacy concerns. Others (National Academies of Sciences, Engineering, and Medicine, 2018) have suggested evaluating community-based health literacy interventions by partnering with other community organizations or people possessing local knowledge and expertise and building trust by developing long-term relationships with community members and institutions. The HTTTYD HANDbook programs conducted by CEAs are uniquely situated to meet this recommendation.

Future implementation of the HANDbook program could be expanded to include role play with simulated providers. Opportunities for participants to practice and demonstrate competencies asking and answering questions and giving their 2-minute histories with “providers” may result in increased engagement and shared decision-making in real clinical settings. Future research on this implementation expansion and its impact is needed.

## Study Limitations

Some limitations apply to the analyses presented. The sample was a convenience sample of relatively homogeneous participants. Caution should be taken concerning generalizing these results to other populations, although the analyzed sample is similar to the study area population. The one-group pre-test and post-test quasi-experimental design can be considered weak and particularly vulnerable to internal validity threats because it has no control group (Portney & Watkins, 2009), although people serve as their own control. Social desirability bias may also affect the results. Participants knew the purpose of the study was to determine changes in confidence communicating with health care providers. They may not have wanted to appear to lack such skills as this may have a negative connotation or stigma associated with low literacy. However, participants were encouraged to respond honestly to the surveys, so health literacy could be accurately assessed. Finally, the study is subject to nonresponse bias as the response rate at the 3-month follow up was 30.5%. However, the demographics in terms of age, gender, and race/ethnicity between respondents and nonrespondents are sufficiently similar to make inferences.

## Conclusion

The National Action Plan to Improve Health Literacy contains seven goals along with several strategies to achieve these goals (Office of Disease Prevention and Health Promotion, 2010). One goal is to increase basic research and the development, implementation, and evaluation of practices and interventions to improve health literacy. Although examples of health literacy programs exist, evidence supporting their effectiveness is inadequate (National Academies of Sciences, Engineering, and Medicine, 2018). The HTTTYD HANDbook program meets the recommendations for successful health literacy programs (National Academies of Sciences, Engineering, and Medicine, 2018) and its effectiveness is demonstrated by the significant positive outcomes noted in this study. The delivery of the HTTTYD HANDbook program by CEAs and trained volunteers to older rural adults demonstrates access to understudied populations (Manafo & Wong, 2012). The CEAs live and work in such communities and are thus trusted members of the community, a key element in successful outreach. CEAs' distribution throughout the nation facilitates the sustainability, scalability, and portability of the delivery of the HTTTYD HANDbook program and provides a framework for successfully recruiting and training community volunteers.

## Figures and Tables

**Table 1:** Participant Demographics (*N* = 548)

	**Pre-Test and Post-Test**		**Pre-Test, Post-Test, and 3-Month Follow Up**	

**Demographic Category^a^**	***n***	**%**	***n***	**%**

Gender				
Female	460	85	146	88
Male	81	15	19	11.4

Medicaid recipients	137	25	46	27.7

Race/ethnicity				
White	362	67	123	74.1
Black	158	29.3	36	21.7
Multiracial/multiethnic	15	2.8	5	3
American Indian	5	0.9	1	0.6
Hispanic	3	0.6	1	0.6

Age				
18–64 years	171	32.1	62	37.3
65+ years	362	67.9	100	60.2

Participants in rural counties	417	76	114	68.7

a Not all participants provided information.

**Table 2:** Mean Test Scores, Points and Directionality of Change, and Sustained Change

	**Mean Scores**			**Points and Directionality of Change**		**Sustained Changes**	

**Item**	**Pre-Test**	**Post-Test**	**3-Month Follow Up**	**Pre-Test to Post-Test (*p* Value)**	**Post-Test to 3-Month Follow-Up (*p* Value)**	**Pre-Test to 3-Month Follow-Up (*p* Value)**	**Effect Size**

Confidence in:							
Being prepared to talk to one's doctor	3.94	4.43	4.43	.000	NS	.000	.41
Telling one's doctor everything necessary to know in a short visit	3.86	4.36	4.38	.000	NS	.000	.39
Repeating back what one's doctor tells them in his or her own words	3.67	4.28	4.04	.000	.030	.001	.26
Taking one's medications as prescribed	4.49	4.63	4.74	.045	NS	.003	.22

Overall scores on How to Talk to Your Doctor HANDbook Program	15.94	17.76	17.61	.000	NS	.000	.43

Note. NS = not significant.
